# Tensionable lesser tuberosity osteotomy repair for anatomic total shoulder arthroplasty

**DOI:** 10.1016/j.xrrt.2023.09.014

**Published:** 2023-11-03

**Authors:** Matthew R. Cohn, William Baker, Corey J. Schiffman, Amar S. Vadhera, Sebastian Bustamante, Luke S. Austin

**Affiliations:** a Department of Orthopaedic Surgery, Rothman Orthopaedic Institute at Thomas Jefferson University Hospital, Philadelphia, PA, USA; b Department of Orthopaedic Surgery, Jefferson Health New Jersey, One Medical Center Drive, Stratford, NJ, USA

**Keywords:** Subscapularis repair, Lesser tuberosity osteotomy, Shoulder arthroplasty, Technique, Anatomic total shoulder arthroplasty, Shoulder replacement

## Abstract

A lesser tuberosity osteotomy (LTO) is commonly performed during total shoulder arthroplasty to access the glenohumeral joint. Healing of the LTO is critical to optimizing the outcome of the procedure and is enhanced by a repair that provides stability and compression across the osteotomy site. The purpose of this article is to describe a technique that uses a tensionable suture construct to repair the LTO during anatomic total shoulder arthroplasty using a stemless humeral component. The technique involves passing a row of high-tensile sutures through bone tunnels lateral to the osteotomy site (transosseous sutures) and another row of sutures through the humeral implant (implant sutures). One limb of each bone tunnel suture is then tied to its corresponding limb of implant suture and the remaining free strands of the tied sutures are manually tensioned and tied to each other. This technique is an efficient and reproducible method for creating compression and stability across the osteotomy site that facilitates bony healing.

The deltopectoral approach is the workhorse exposure for anatomic total shoulder arthroplasty (TSA), which involves takedown of the subscapularis tendon to access the glenohumeral joint. Techniques to release the subscapularis include lesser tuberosity osteotomy (LTO), which maintains the subscapularis tendon to its bony insertion; subscapularis peel, in which the subscapularis is released from its insertion on the lesser tuberosity; and subscapularis tenotomy, which uses an intratendinous split of the subscapularis tendon. Several comparative trials have evaluated outcomes after each technique and have not indicated a clear functional benefit of one technique over the others.^1^^,^^13^, ^14^, ^15^, ^16^ However, some surgeons prefer LTO due to theoretical benefits of bone-to-bone healing, superior subscapularis-specific physical examination findings, and the ability to monitor for LTO failure radiographically (Fig. 1, *A*) and via ultrasound.^4^^,^^5^^,^^16^ In addition, LTO may improve glenoid exposure in challenging cases.

**Figure 1:**
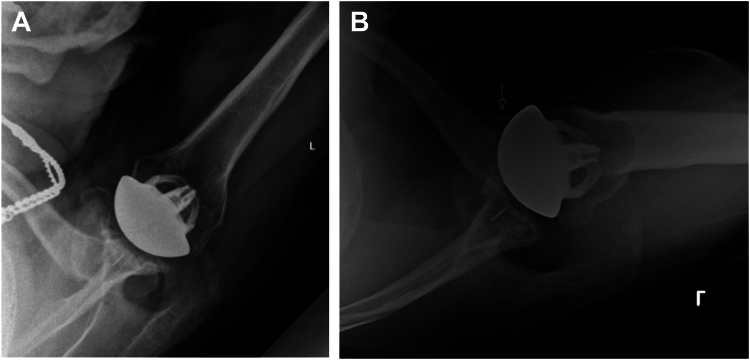
(**A**) Healed lesser tuberosity repair at 6 weeks postoperatively. (**B**) Lesser tuberosity repair failure (indicted by arrow) at 6 weeks postoperatively.

Regardless of takedown technique, subscapularis failure is a feared complication following anatomic TSA with an incidence ranging from 1% to 6% (Fig. 1, *B*).^2^^,^^19^^,^^24^ Failure may lead to pain, functional internal rotation weakness, and anterior subluxation that risks edge-loading and loosening of the glenoid component.^3^^,^^10^ Therefore, achieving a stable subscapularis repair that facilitates healing is critical to the success of the procedure.

Several methods for LTO repair have been described and have shown positive clinical, radiographic, and biomechanical results.^6^^,^^7^^,^^9^^,^^11^^,^^12^^,^^17^^,^^21^ Key elements of repair include generating sustained compression, minimizing suture pull-out, and avoidance of gapping at the osteotomy site. Optimal repair techniques should achieve these goals while also remaining technically efficient and reproducible.^20^ While several LTO repair techniques have been described previously, few incorporate these characteristics while remaining simple and reproducible with fixation to both the native bone and implant. The technique presented in this article uses high-tensile strength sutures passed around the osteotomy bed as well as sutures through the implant that are tied in a tensionable manner to generate compression across the osteotomy site.

## Surgical technique

### Lesser tuberosity osteotomy

**Figure 2:**
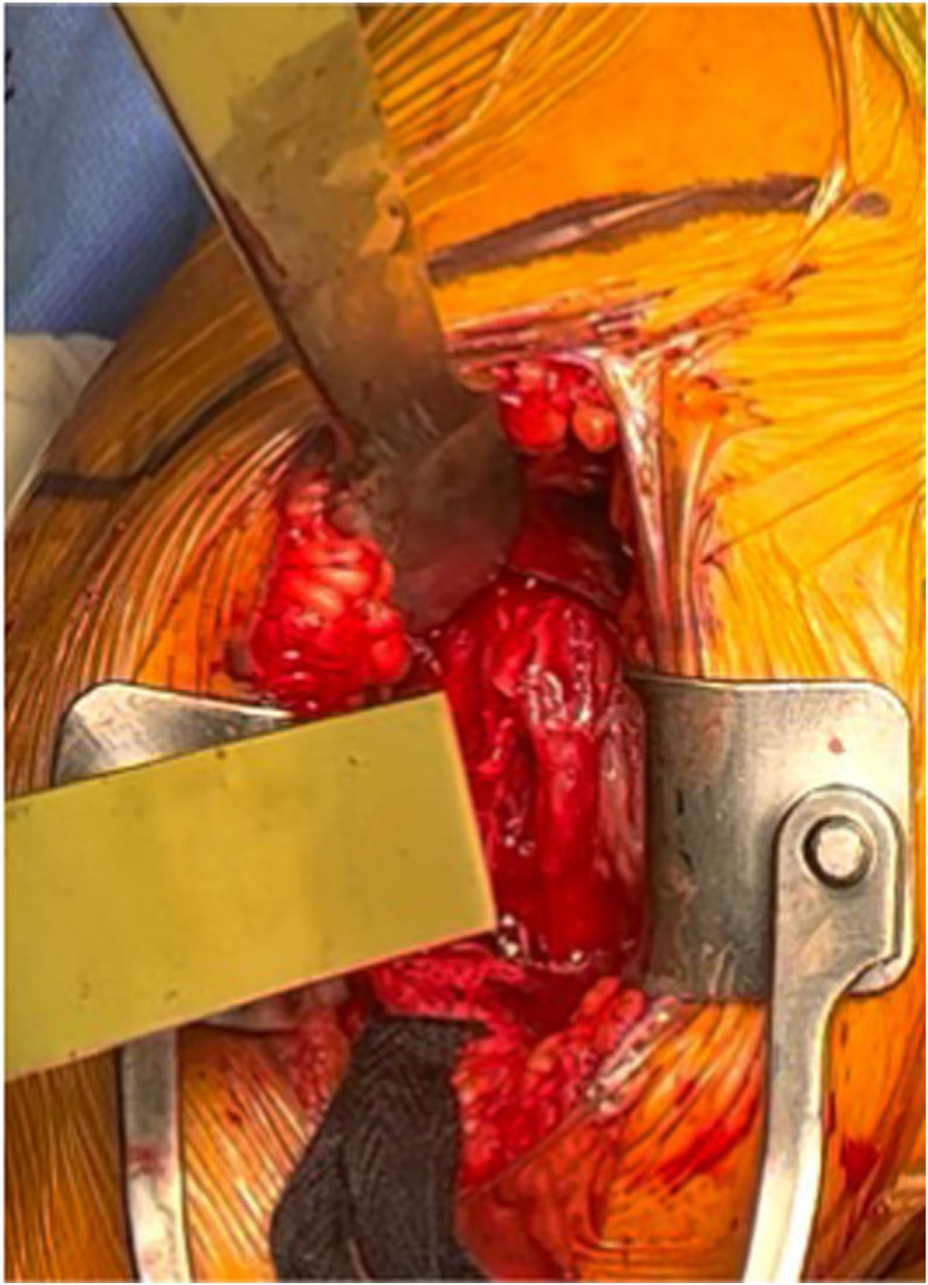
Author’s preferred curved osteotome to perform lesser tuberosity osteotomy.

**Figure 3:**
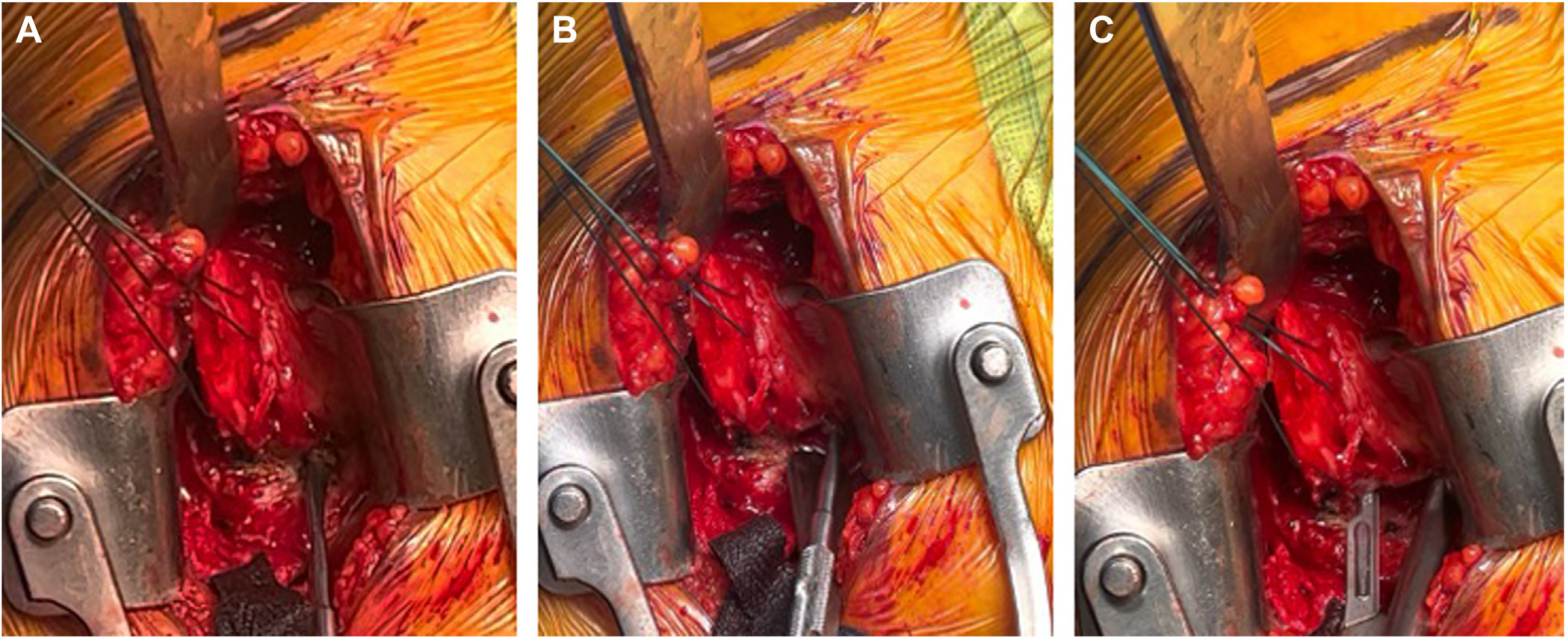
(**A**-**C**) Using a freer followed by a cobb, the interval between the subscapularis and anterior capsule is defined. A15 blade from medial to lateral is used to free the subscapularis from the underlying capsule to allow excellent subscapularis mobilization and complete anterior capsulectomy.

The patient is placed in a beach chair position with the head of the bed elevated to 45° from the horizontal. A standard deltopectoral approach is performed, and the long head biceps tendon is tenodesed to the pectoralis major tendon. A large curved osteotome is used to perform the LTO. The curve of the osteotome is pointed medially with the arm in internal rotation (Fig. 2). With the osteotome held parallel to the insertion of the subscapularis, the osteotomy is aimed to exit between the medial edge of the subscapularis tendon and the cartilaginous edge of the humeral head, to remove 0.5 cm to 1 cm thickness of bone. A sagittal saw may also be used to perform an osteotomy, although we prefer an osteotome for efficiency and theoretically less risk of thermal necrosis of the osteotomy bed. Next, 2 #2 Ethibond traction sutures (Ethicon; Raritan, NJ, USA) are placed around the osteotomized lesser tuberosity to help maintain control of the lesser tuberosity throughout the case. Electrocautery is used to free the inferior edge of the subscapularis so that a freer elevator can be used to develop the interval between the subscapularis musculature and the anterior capsule. After this interval is developed, the freer elevator is exchanged for a small cobb elevator, which widens the interval between the subscapularis and anterior capsule. The Ethibond sutures are lifted away from the patient to place the subscapularis on tension, and a 15 blade is used to release the subscapularis from the anterior capsule to fully mobilize the musculotendinous subscapularis (Fig. 3, *A*-*C*). The anterior capsule is excised later in the case during the glenoid exposure. We prefer to perform a capsulotomy in every case, as evidence suggests that the anterior capsule is pathologic in osteoarthritis and may be associated with posterior glenoid wear, humeral head subluxation, and stiffness.^18^ In addition, resecting the anterior capsule allows for better excursion of the subscapularis for repair and improves glenoid exposure in our experience. Following the LTO and humeral exposure, osteophytes are excised to identify the native articular margin and a freehand humeral cut is made at the articular margin so native anatomy may be recreated with the humeral implant. No specific modifications to the humeral head cut are necessary to optimize the LTO and repair.

### Repair preparation

**Figure 4:**
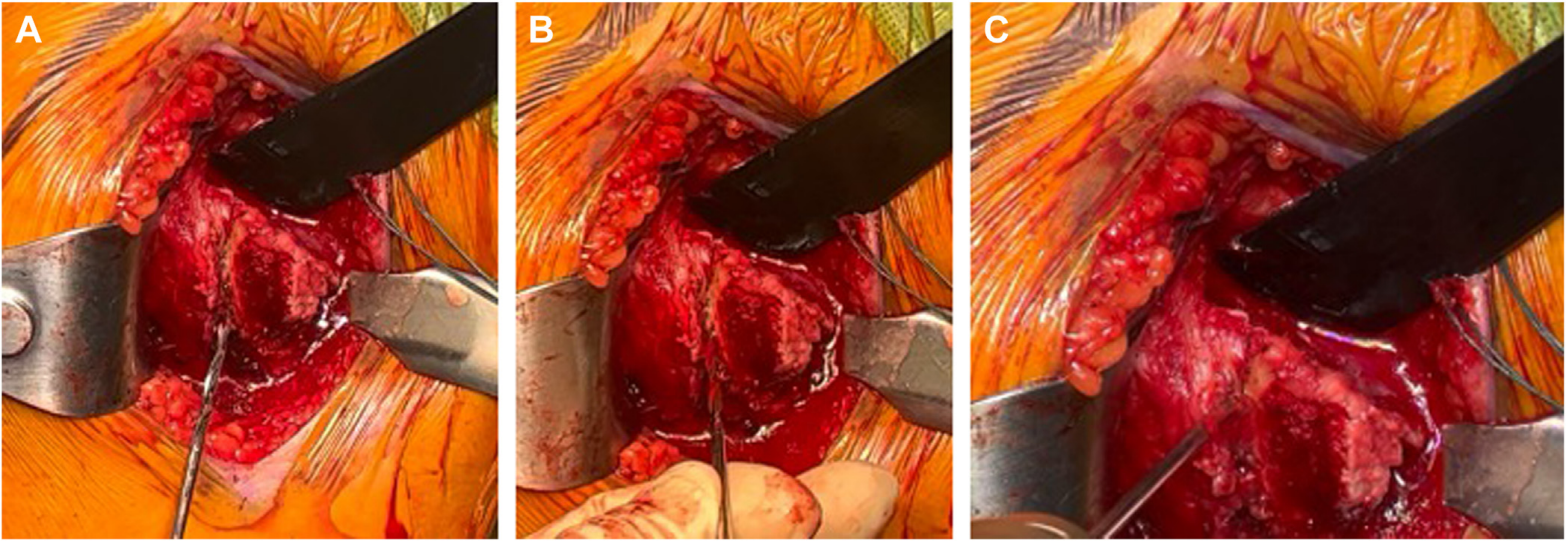
(**A**-**C**) Placement of drill holes within the intertubercular groove. Most distal drill hole is about 1 cm distal to the most superior aspect of the groove, just proximal to the pec major as it runs across the humerus to insert laterally on the humerus. The next 2 drill holes are separated equally toward the superior most aspect of the groove.

**Figure 5:**
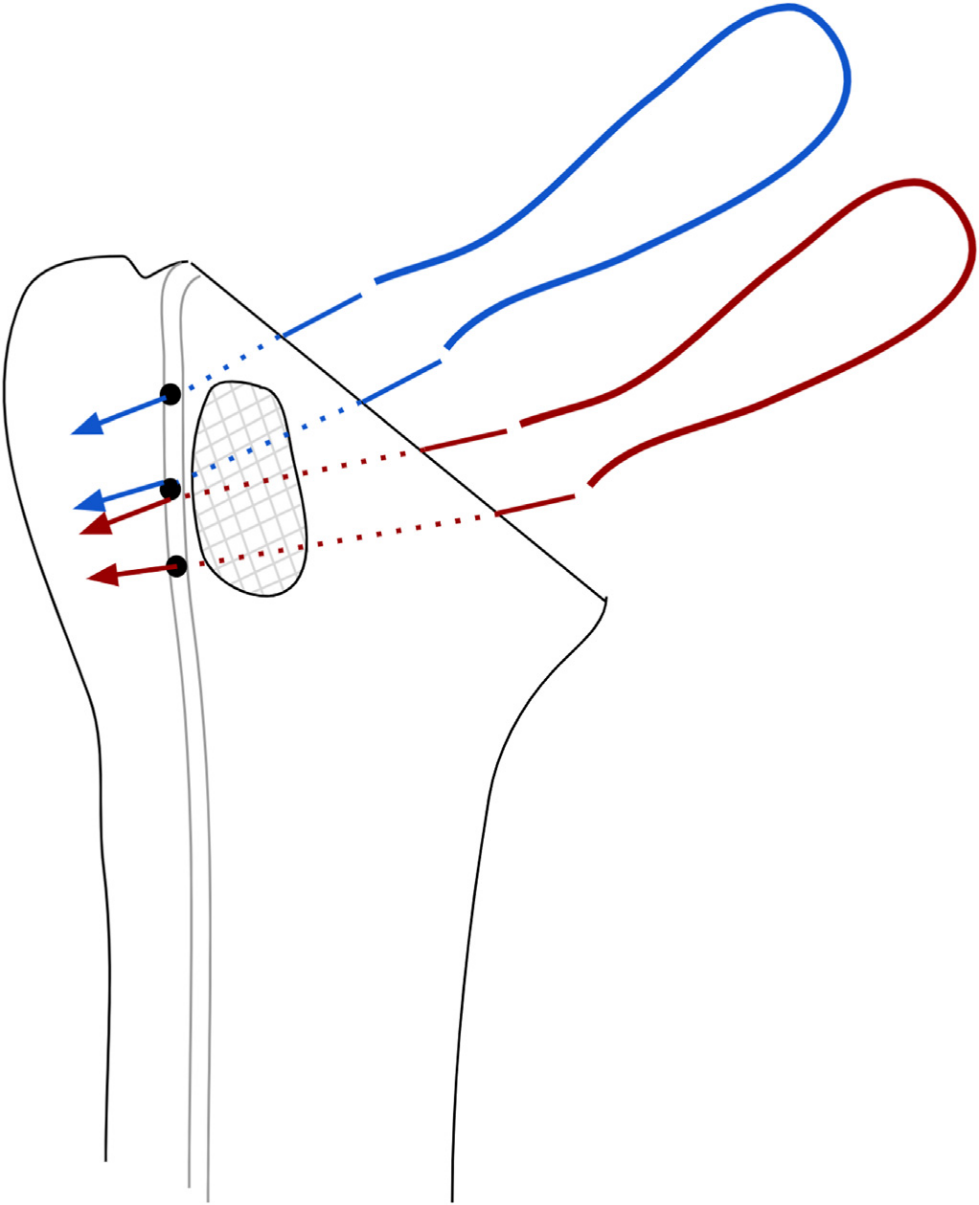
Two Ultratapes (Smith and Nephew, Watford, England) are passed through 3 transosseous drill holes that are created from the bicipital groove to the humeral osteotomy site. One limb from the superior suture (blue) is passed through the most superior hole and one limb from the inferior suture (red) is passed through the inferior hole with a Hewson suture passer. The remaining limbs from each suture are then both passed together through the Middle hole.

The preparation of the glenoid and humerus is then completed based on surgeon preference. Prior to implant insertion, the arm is placed into internal rotation to present the bicipital groove. Three drill holes for suture passage (“transosseous sutures”) are placed along lateral edge of the bicipital groove, lateral to the osteotomy site, proximal to the insertion of the pectoralis major insertion, and exiting in the prepared metaphysis (Fig. 4, *A*-*C*). The bicipital groove bone is the strongest in the proximal humerus and drill holes are typically separated by 1 cm from inferior to superior, with the most proximal drill hole at the proximal edge of the osteotomy bed and the distal drill hole at the most distal aspect. This separation is important to provide an adequate bone bridge to ensure no suture pull-out. In large patients, we will occasionally add a fourth drill hole and a third suture. Using a Hewson suture passer, one limb of an Ultratape (Smith and Nephew; Watford, England), a suture size equivalent to the traditional #2 suture, is pulled through the inferior most drill hole from medial to lateral. A second Ultratape is then passed through the superior-most hole in the same fashion. The 2 remaining limbs of each are then passed through the middle hole medial to lateral and the pairs of suture limbs are secured with a hemostat (Fig. 5).

**Figure 6:**
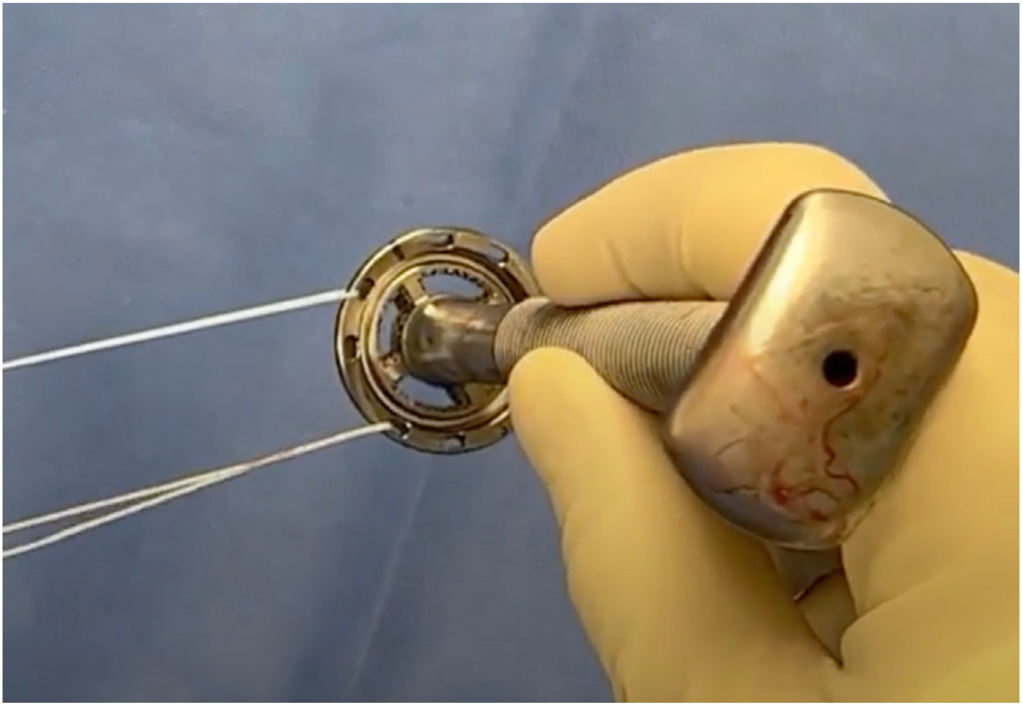
Two high-tensile sutures are passed through the humeral component in the 11 o’clock and 8 o’clock positions.

On the back table, 2 Ultratape sutures are placed through anterior holes in the humeral component (“implant-sutures”). Our preferred stemless humeral component (Inhance; Ignite Orthopedics LLC; Warsaw, IN, USA) contains holes that may be oriented in 11 o’clock and 8 o’clock positions for sutures passage (Fig. 6). Although this is the author’s preferred implant, this LTO compression repair technique can be applied to any stemmed or stemless component with suture holes along the anterior rim of the humeral stem. With the humeral implant held in its planned position over the humerus, electrocautery is used to mark the bone at the corresponding 11 and 8 o’clock positions on the humerus. A Leksell rongeur is used to remove a small amount of cancellous bone at the marked locations on the bone to create space for the implant sutures to slide freely. The humeral component and implant sutures are placed into the desired position and the sutures are checked to ensure that they slide freely. The humeral head is then impacted into position.

### Suture repair

**Figure 7:**
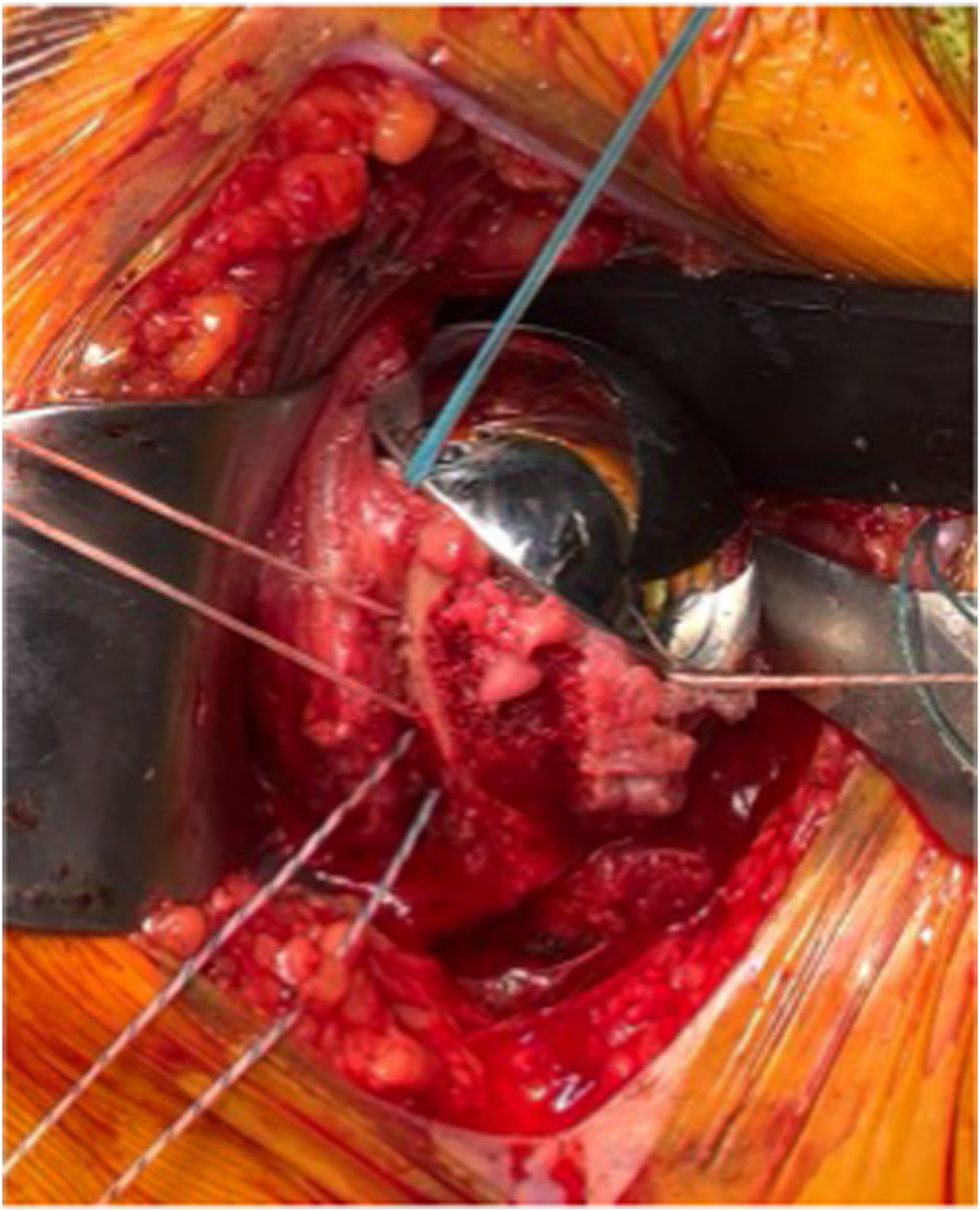
Sutures arrangement following humeral implantation. Two medial sutures are passed through the stemless implant and 2 lateral sutures are passed through transosseous tunnels in the biceps groove.

**Figure 8:**
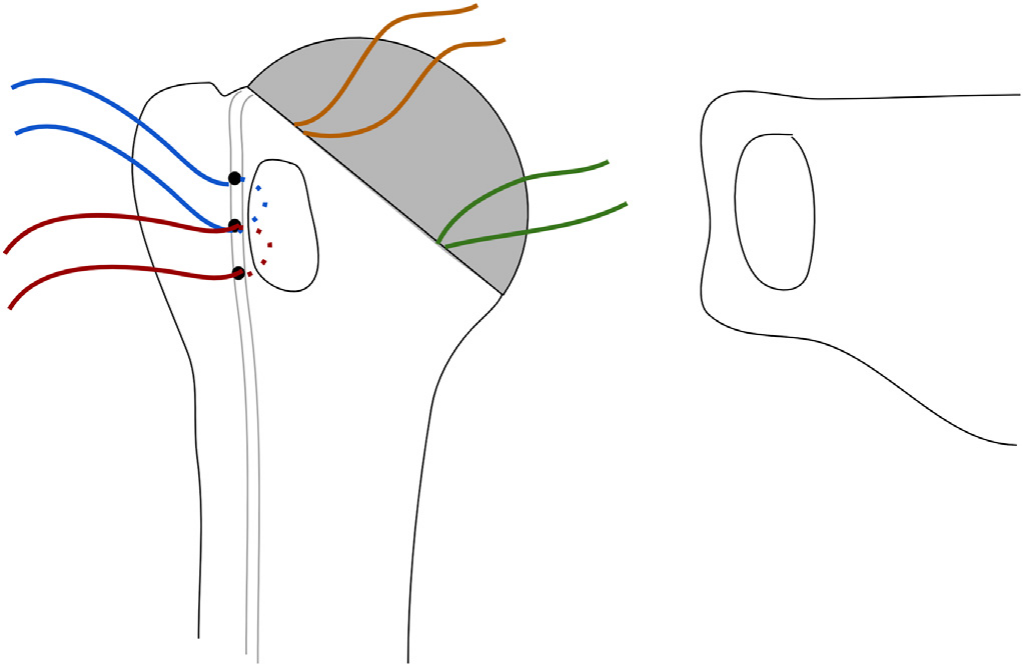
After the humeral component is placed, there are 4 sutures in place. One through the 11 o’clock hole in the implant (orange) and another through the 8 o’clock hole (green). Two transosseous sutures (red and blue) remain passed through the drill holes in the bicipital groove. The orange and green implant sutures will be passed through the subscapularis tendon (Right) just medial to the lesser tuberosity bone block.

**Figure 9:**
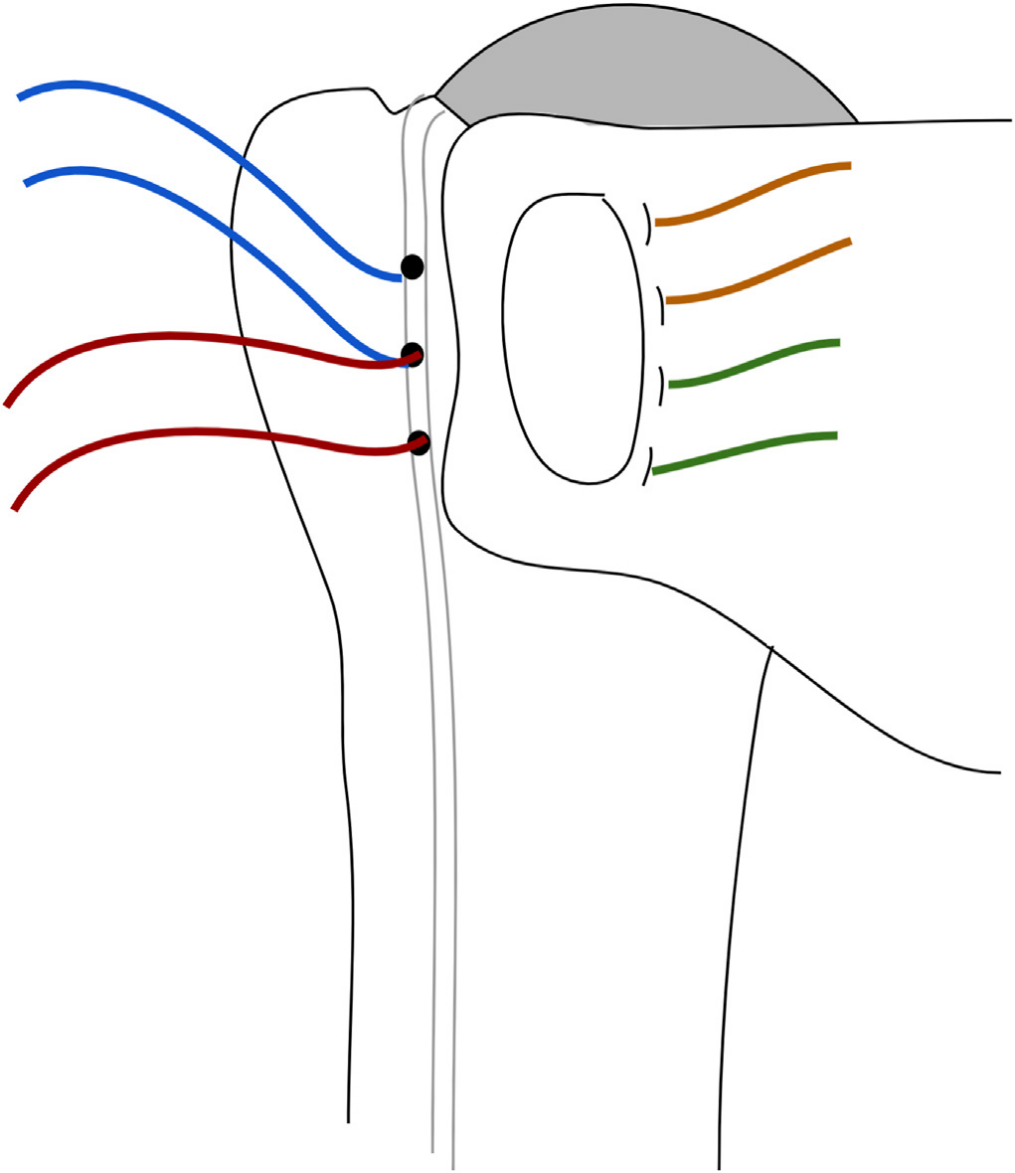
All 4 limbs of the implant sutures (orange and green) are then passed just medial to the lesser tuberosity bone block, spanning the entirety of the osteotomy.

Once the humeral component is placed, there are 4 sutures present (2 transosseous sutures and 2 implant sutures within the phalanges of the component; Figs. 7 and 8). To begin the repair, a free needle is used to pass the 4 limbs from the 2 implant sutures through the subscapularis at the bone-tendon junction, just medial to the osteotomized lesser tuberosity. A free needle is used to pass each limb through the subscapularis tendon moving from superior to inferior. It is important to spread these sutures evenly in the subscapularis osteotomy tissue and immediately medial to the bone block to allow for better compression in the end repair (Fig. 9).

**Figure 10:**
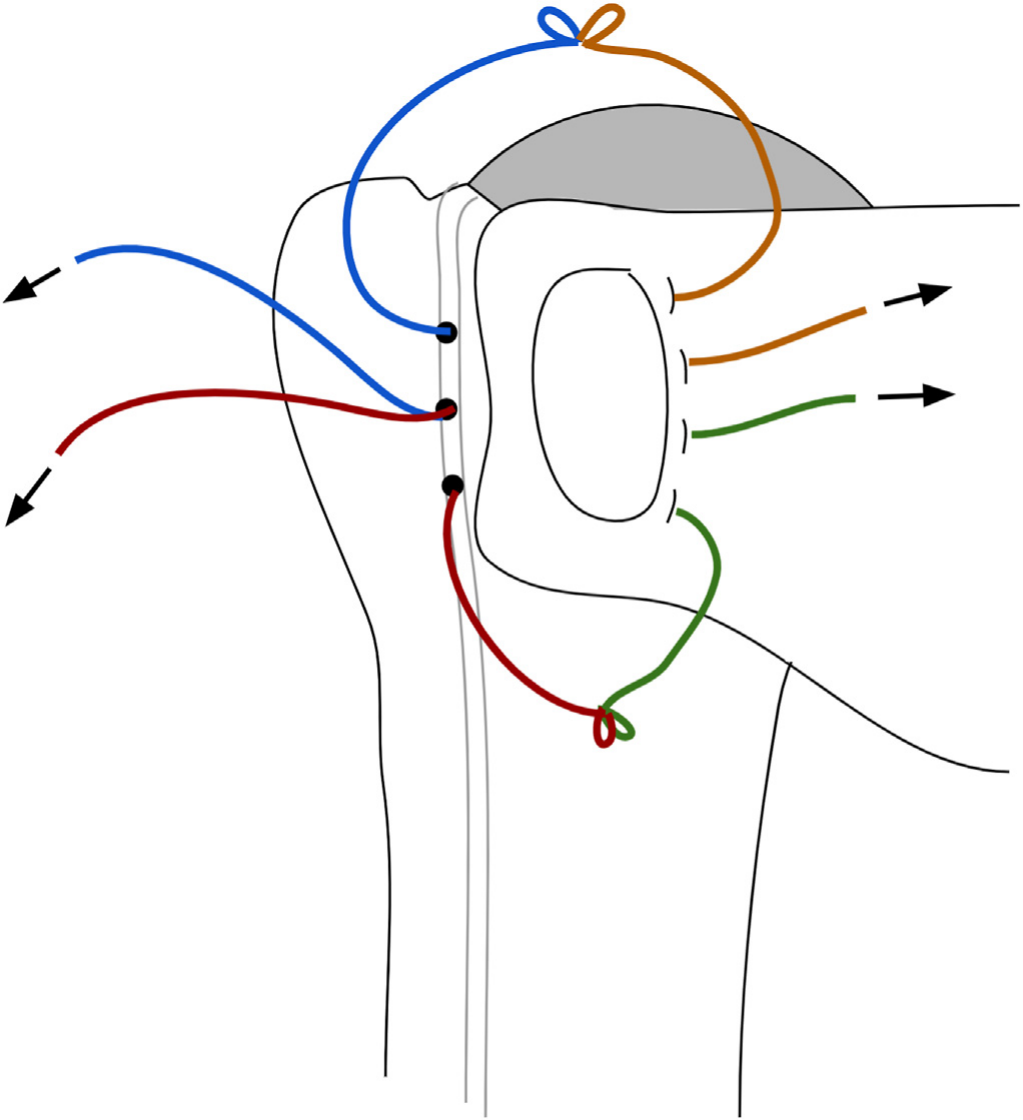
The most superior transosseous (blue) and implant suture (orange) limbs are tied end-to-end and are backed up by 2-handed square knots. Pulling up on the free suture limbs will bring the knots tightly against the lesser tuberosity osteotomy, generating compression. The free limbs (blue and orange) are tensioned and tied using a surgeon’s knot and followed by 2-handed square knots. This step is repeated with the inferior suture limbs (red and green).

**Figure 11:**
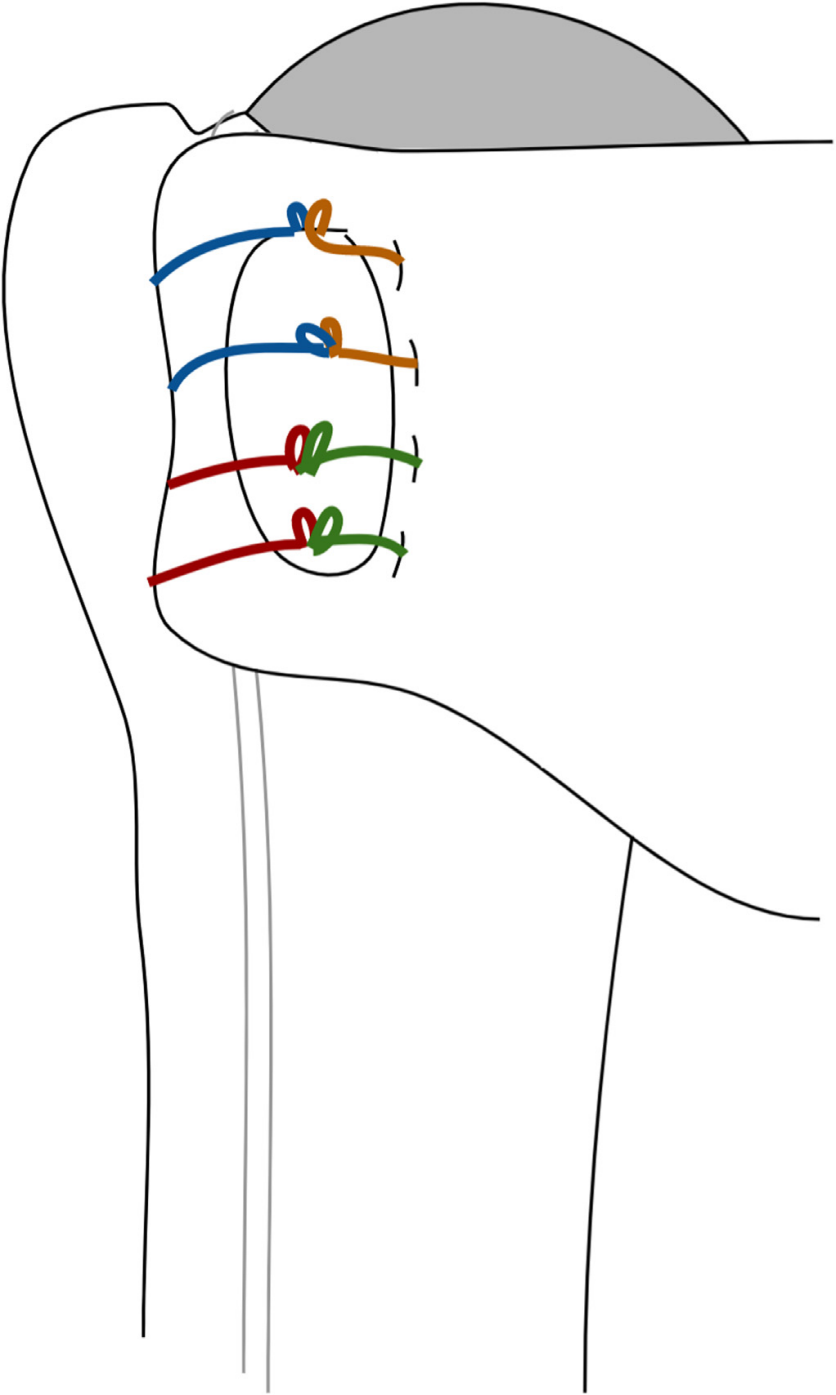
The remaining suture limbs are tensioned and tied, generating compression across the osteotomy site.

**Figure 12:**
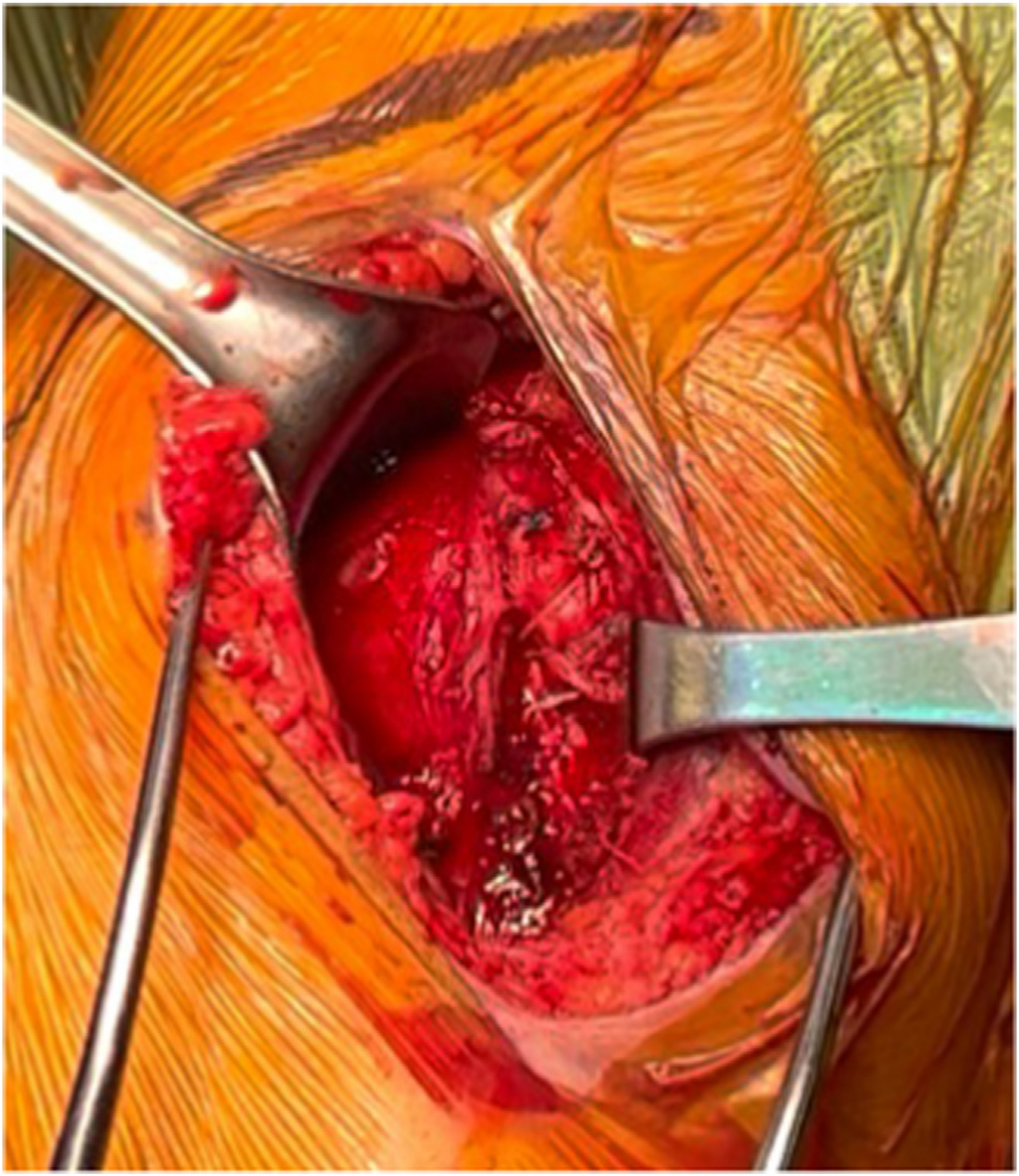
Final repair construct.

Next, the suture limb that was passed through the most superior drill hole in the bicipital groove is tied to the most superior implant suture that was passed through the subscapularis tendon using an end-over-end tie as shown in the technique video (Supplementary Video S1). A series of 2-handed square knots are then performed to secure the knot in place and the strands are cut with a 0.5 cm tail. While holding the free limb of the superior transosseous suture (blue in Figs. 5, Figure 8, Figure 9, Figure 10, Figure 11) in one hand and the free limb of the 11 o’clock implant suture (orange) in the other hand, the sutures are tensioned, bringing the previously tied knot to lay flat against the superior aspect of the subscapularis tendon and applying compression over the osteotomy repair (Fig. 10). With this technique, the tension applied to the free limbs becomes compression over the osteotomy site. These 2 free limbs are then tied to each other over the osteotomy using a 2-handed surgeon’s knot, followed by 3 square knots. These steps are then repeated with inferior set of transosseous and implant sutures (Fig. 11). A #2 FiberWire (Arthrex, Naples FL, USA) is routinely used at this point to close the rotator interval in a figure-of-eight manner to reinforce the final repair (Fig. 12).

## Discussion

The LTO repair technique presented in this paper involves tying laterally-based transosseous sutures to medially-based implant sutures, bridging the osteotomy and providing compression. In addition, by tying 2 pairs of sutures end-to-end and pulling the free limbs through drill holes in the bicipital groove, the construct is tensionable, allowing the surgeon to control the compression across the LTO site, while providing fixation to both the native proximal humerus and the humeral component.

While subscapularis peel and tenotomy have shown generally comparable functional outcomes compared to LTO,^15^^,^^16^ a systematic review of 14 studies suggested that LTO provides the highest healing rate.^4^ Approximately 93% of LTO repairs healed based on computed tomography, magnetic resonance imaging, or ultrasonography compared to 84% of peel repairs and 76% of tenotomy repairs.^4^ In addition, patients who underwent LTO demonstrated higher rates of normal abdominal compression and lift off tests (79% and 81%, respectively) compared to subscapularis tenotomy (67% and 66%, respectively). Levine et al^15^ performed a randomized controlled trial that compared outcomes of LTO and tenotomy among 59 patients undergoing stemmed anatomic TSA. The authors found no differences in clinical outcomes or range of motion at 1-year follow-up. However, the healing rate of LTO was found to be 93% based on axillary radiographs compared to a tenotomy healing rate of 87% based on ultrasound. Aibinder et al^1^ performed a retrospective study that compared LTO, peel, and tenotomy techniques among 188 patients who underwent anatomic TSA with a stemless implant. At 2-year follow-up, pain, American Shoulder and Elbow Surgeons scores, and patient-reported instability were comparable among the 3 repair techniques. Subscapularis failure requiring reoperation occurred in 2 patients in the peel group, whereas no failures were seen with LTO or tenotomy.^1^

To maximize the chance of bone-to-bone healing, it is important to achieve stable time-zero fixation and compression. Several biomechanical studies have evaluated the strength of various LTO repair techniques. Two common constructs for LTO repair are tension-band and compression techniques. The tension-band technique described by Gerber et al^8^ involves horizontal mattress sutures passed medial to the LTO that travel over the subscapularis tendon and through bone tunnels from the bicipital groove to exit more laterally from the greater tuberosity. In contrast, the compression technique involves sutures that completely encircle the LTO fragment.^7^^,^^12^^,^^21^

A variety of compression techniques have been reported, all of which use sutures around the LTO that are passed laterally through bone tunnels adjacent to the LTO bed and medially through bone tunnels or the resection surface of the humeral head.^7^^,^^12^^,^^21^ The technique presented in this paper applies a compression technique, but instead of encircling the osteotomy bed, the sutures are anchored medially to the implant and laterally by bone tunnels. A biomechanical comparison of compression and tension-band techniques was performed by Heckman et al, which demonstrated that a compression dual-row repair had greater ultimate tensile strength (632N) and lower initial displacement (4.6 mm) compared to tension-band repair (511N and 6.9 mm). Previously described techniques typically involve wrapping sutures around the humeral stem, which is not possible with a stemless implant.^6^^,^^11^^,^^17^ Others have described passing repair sutures through the stemless implant in such a manner that does not allow the sutures to slide.^23^ The current technique is the first to our knowledge that uses fixation medially to suture holes of the implant and allows tensioning of the sutures in a sliding fashion to provide excellent compression.

Biomechanical data also suggest that LTO repair in which the sutures are wrapped around the humeral stem improves time-zero stability.^12^^,^^22^ As stemless implants become increasingly popular, surgeons may adapt their repair technique to secure the LTO to the stemless humeral component. In the presented technique, sutures are passed through peripheral holes of the stemless implant medially to anchor the repair. Theoretically, this may be advantageous to passing sutures through the bed of the humeral osteotomy site, where sutures may loosen if they pull through relatively weak cancellous bone or abrade against the rough surface of the metaphysis. Although we describe this technique with a stemless implant, it can be used with stemmed implants that contain holes for sutures passage as well.

## Conclusion

Maximizing stability of an LTO repair depends on secure fixation of sutures to the proximal humerus and/or humeral implant and generation of compression across the osteotomy site. In the presented technique, sutures are secured to the implant medially and transosseous tunnels in the bicipital groove laterally in such a manner that is tensionable, allowing for maximal compression of the repair. This novel technique for tensioning across the repair bed allows for immediate axial compression across the LTO, which theoretically should increase the strength of the repair to permit bone-to-bone healing. Additionally, this repair is simple and reproducible and provides fixation to both the native proximal humerus and the humeral implant. Future biomechanical studies should evaluate the load to failure with this construct compared to other previously described techniques. Additionally, clinical studies should evaluate the rate of bony union and subscapularis failure compared to other subscapularis repair methods in effort to find the optimal repair technique.

## Disclaimers:

Funding: No funding was disclosed by the authors.

Conflicts of interest: Luke S. Austin, MD: DePuy, A Johnson & Johnson Company: IP royalties; Paid consultant. Ignite Orthopedics: IP royalties; Paid consultant; Stock or stock Options Preliminary patent filed for reference implant: Stock or stock Options Rothman Institute and Related Holdings: Other financial or material support Zimmer: Research support. The other authors, their immediate families, and any research foundation with which they are affiliated have not received any financial payments or other benefits from any commercial entity related to the subject of this article.

The editor making the decision to accept this paper for publication had no conflicts of interest related to the decision. Further, peer review of this paper was handled independently of any author of this paper.
